# An Ultrasensitive Biosensor for Detection of Femtogram Levels of the Cancer Antigen AGR2 Using Monoclonal Antibody Modified Screen-Printed Gold Electrodes

**DOI:** 10.3390/bios11060184

**Published:** 2021-06-07

**Authors:** Wioleta Białobrzeska, Karolina Dziąbowska, Małgorzata Lisowska, M. Aiman Mohtar, Petr Muller, Borivoj Vojtesek, Radovan Krejcir, Robert O’Neill, Ted R. Hupp, Natalia Malinowska, Ewelina Bięga, Daniel Bigus, Zofia Cebula, Katarzyna Pala, Elżbieta Czaczyk, Sabina Żołędowska, Dawid Nidzworski

**Affiliations:** 1 Institute of Biotechnology and Molecular Medicine, 3 Trzy Lipy St., 80-172 Gdansk, Poland; n.malinowska@ibmm.pl (N.M.); e.biega@ibmm.pl (E.B.); d.bigus@ibmm.pl (D.B.); z.cebula@ibmm.pl (Z.C.); sabina.zoledowska@etongroup.eu (S.Ż.); dawid@etongroup.eu (D.N.); 2 SensDx, 14b Postępu St., 02-676 Warszawa, Poland; karolina.dziabowska@gmail.com (K.D.); katarzyna.pala@etongroup.eu (K.P.); ela@etongroup.eu (E.C.); 3 International Centre for Cancer Vaccine Science, University of Gdansk, Kładki 24 St., 80-822 Gdańsk, Poland; malgorzata.lisowska@ug.edu.pl (M.L.); ted.hupp@ed.ac.uk (T.R.H.); 4 UKM Medical Centre, UKM Medical Molecular Biology Institute (UMBI), Universiti Kebangsaan Malaysia, Cheras, Kuala Lumpur 56000, Malaysia; m.aimanmohtar@ppukm.ukm.edu.my; 5 Research Centre for Applied Molecular Oncology, Masaryk Memorial Cancer Institute, 65653 Brno, Czech Republic; pmuller@post.cz (P.M.); vojtesek@mou.cz (B.V.); radovan.krejcir@mou.cz (R.K.); 6 Cambridge Oesophagogastric Centre, Cambridge University Hospitals NHS Foundation Trust, Cambridge CB2 0QQ, UK; robertoneill@nhs.net; 7 Institute of Genetics and Molecular Medicine, University of Edinburgh, Edinburgh EH4 2XR, UK

**Keywords:** AGR2 protein, sensor, screen-printed gold electrode, electrochemical impedance spectroscopy

## Abstract

The detection of cancer antigens is a major aim of cancer research in order to develop better patient management through early disease detection. Many cancers including prostate, lung, and ovarian secrete a protein disulfide isomerase protein named AGR2 that has been previously detected in urine and plasma using mass spectrometry. Here we determine whether a previously developed monoclonal antibody targeting AGR2 can be adapted from an indirect two-site ELISA format into a direct detector using solid-phase printed gold electrodes. The screen-printed gold electrode was surface functionalized with the anti-AGR2 specific monoclonal antibody. The interaction of the recombinant AGR2 protein and the anti-AGR2 monoclonal antibody functionalized electrode changed its electrochemical impedance spectra. Nyquist diagrams were obtained after incubation in an increasing concentration of purified AGR2 protein with a range of concentrations from 0.01 fg/mL to 10 fg/mL. In addition, detection of the AGR2 antigen can be achieved from cell lysates in medium or artificial buffer. These data highlight the utility of an AGR2-specific monoclonal antibody that can be functionalized onto a gold printed electrode for a one-step capture and quantitation of the target antigen. These platforms have the potential for supporting methodologies using more complex bodily fluids including plasma and urine for improved cancer diagnostics.

## 1. Introduction

Anterior Gradient-2 (AGR2) was identified as a key protein involved in the assembly of the dorso–anterior ectoderm that forms the cement gland and maintains forebrain integrity [1,2]. AGR2 has since been shown to be an endoplasmic reticulum (ER) localized protein disulfide isomerase superfamily member [3] that is upregulated in a large number of human cancers [4,5]. The cancer associated functions of AGR2 have been inferred from several lines of research including (i) AGR2 drives cement gland production, whose function promotes the attachment of the growing epithelium to a solid support [6], (ii) AGR2 can mediate metastatic growth in cancer models [7], and (iii) AGR2 can mediate limb regeneration in amphibia [8]. AGR2 has also been implicated in a diverse range of diseases including asthma [9] and inflammatory bowel disease [10].

AGR2 is over-expressed in a diverse set of human cancers including breast [11], prostate [12], pancreatic [13], liver [14], ovarian [15], esophagus [16,17,18], and lung cancers [19]. In some cases, AGR2 is secreted allowing remodeling of the pro-metastatic niche [20,21]. Accordingly, mass spectrometry and ELISA methodologies have been used to detect AGR2 peptide fragments in urine or plasma [22,23,24,25,26]. In addition to this diagnostic potential of AGR2, inhibition of extracellular AGR2 function using antibodies or peptides suggests therapeutic tools can be developed [27,28,29]. The range of AGR2 concentrations associated with cancer has been described by Edgell et al. [26]. The paper shows significantly increased concentrations of AGR2 protein in plasma from cancer patients relative to normal controls. Plasma AGR2 concentrations were highest in stages II and III ovarian cancer patients and were similarly elevated in patients with both serous and non-serous tumors. The identification of elevated plasma concentrations of AGR2 may provide a useful biomarker to aid in the discrimination of normal and ovarian cancer patients particularly.

In order to develop new diagnostic assays for AGR2, we had developed a panel of monoclonal antibodies or aptamers that can detect AGR2 and its orthologue AGR3 [15]. These antibodies were functionalized using fluorescent conjugation to develop a two-site ELISA that measures the capture of dimeric AGR2 [30,31]. However, these mass spectrometry approaches and ELISA approaches are limited for rapid diagnostics; the mass spectrometry assay requires significant instrumentation time, costs, and sample preparation whilst the ELISA is a two-step process involving antigen capture and antigen detection.

A novel diagnostic platform has been developed using a gold biosensor that enables the specific detection of a specific antigen at ultralow concentrations using a ‘one-step’ methodology; a screen-printed gold electrode is surface functionalized with the antibodies of interest and after the absorption of the target antigen, a change in the electrochemical impedance spectra is measured as a function of different features with a limit of detection of 9.3 cfu/mL defined for a bacterial biomarker [32]. In this report, we aimed to use a gold electrode platform [33] to determine whether the AGR2 antigen can be detected in a ‘one-step’ process from aqueous samples using the monoclonal antibody validated in clinical tissue using immunohistochemistry [15] and in a two-site ELISA assay [31]. Our data demonstrate that the gold electrode can detect femtogram levels of AGR2 protein in vitro cell culture systems and highlights a platform for further evaluation of AGR2 in liquid biopsies.

## 2. Materials and Methods

### 2.1. Reagents and Materials

For the preparation and modification of electrodes, 99.8% Ethanol and Sulfuric Acid were provided by Chempur (Piekary Śląskie, Poland); Phosphate-Buffered Saline (PBS) tablets, Tris Buffered Saline (TBS), 97% 4-ATP, 25% GA, and Bovine Serum Albumin (BSA) were provided by Sigma Aldrich (Munich, Germany).

### 2.2. Electrochemical Procedures

The cyclic voltammetry and electrochemical impedance spectroscopy (EIS) were conducted using a Palmsens4 potentiostat/galvanostat system (PalmSens, Houten, The Netherlands) in the standard three electrode configuration. Gold screen-printed electrodes (DropSens, Asturias, Spain) were used as working electrodes, the Pt mesh was used as a counter electrode, while Ag/AgCl/0.1 M KCl was used as a reference electrode. All the electrochemical tests were carried out in 1 mM K_3_[Fe(CN)_6_]/K_4_[Fe(CN)_6_]/0.1 M PBS that was previously deaerated. CV data were collected in the voltage window of −0.65 to +0.75 V at the scan rate of 100 mV/s, always in triplicate.

In case of the electrochemical impedance spectroscopy measurements (EIS), the frequency ranged from 100 kHz to 0.1 Hz with 50 points. The amplitude of the AC signal was 10 mV. Obtained impedance spectra were recorded at the redox reaction formal potential (EF). EF value was calculated based on the redox peaks’ positions present on the CV voltammograms for the screen-printed electrode and approximately equaled 150 mV. Each potential was held constant for 60 s before each measurement to obtain a steady-state condition. Obtained data were subjected to the analysis using an EIS Spectrum Analyzer according to the proposed electric equivalent circuit (EEQC).

### 2.3. Biomaterials Preparation and Identification by Reference Methods

AGR2 protein was purified as reported previously [18]. A549 cells were grown, and lysates made as described previously (see Supplementary, Figure S1) [34]. The AGR2-monoclonal antibody was purified using Protein A columns and stored in PBS at a temperature of 4 °C, as described previously [15,31]. The incubation time of the electrode with the target solution was 5 min for both the positive and negative samples. The H1299 non-small cell lung carcinoma cell line was from ATCC^®®^, Manassas, VA, USA—CRL-5803™. The cells were maintained in standard culture conditions (37 °C, humidified atmosphere and 5% CO_2_) in high glucose Dulbecco’s modified Eagle’s medium (DMEM; Sigma-Aldrich, St. Louis, MO, USA) supplemented with 10% fetal bovine serum (Thermo Fisher Scientific, Waltham, MA, USA), 1% sodium pyruvate (Sigma-Aldrich), and a penicillin/streptomycin antibiotic mixture (Biosera, Nuaille, France).

### 2.4. Preparation of the Immunosensor

**Figure 1:**
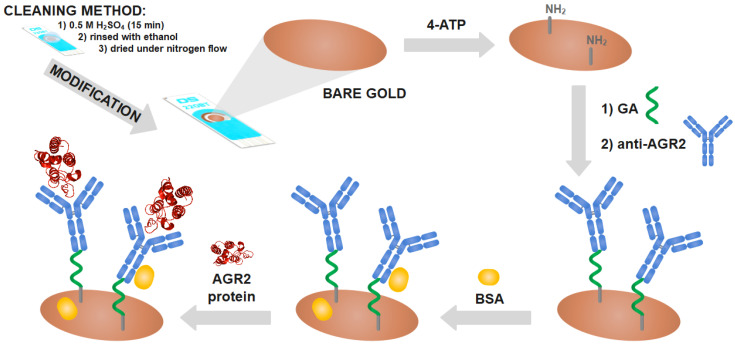
Schematic stages of modified screen-printed gold electrodes. Where 4-ATP stands for 4-aminothiophenol, GA is for glutaraldehyde, anti-AGR2 is for the AGR2 antibody, and BSA is for bovine serum albumin.

The gold electrodes were cleaned with 0.5 M H_2_SO_4_ for 15 min. After each treatment, the gold substrates were rinsed with ethanol and dried under nitrogen flow. Then, the pre-treated gold electrodes were immersed in 0.1 M 4-aminothiophenol in ethanol solution for 12 h in order to form a self-assembled monolayer (SAM). The substrates were then rinsed with ethanol in order to remove the unbonded thiols. To allow antibodies to attach, the thiol-modified electrodes were treated with 2.5% glutaraldehyde for 15 min in a dark place. Next, the gold electrodes were rinsed with water and dried under nitrogen and 10 µg/µL of the anti-AGR2 IgG was dropped onto the surface at 37 °C for 1 h. The excess antibodies were removed by rinsing with PBS. Then, the antibody-modified electrodes were treated with 0.1% BSA for 30 min, to block the unreacted and non-specific sites. After rinsing with PBS and water, the electrodes were dried under nitrogen. The mechanism for this activation can be seen in Figure 1.

## 3. Results

### 3.1. Characterization of the Modified Electrode

**Figure 2:**
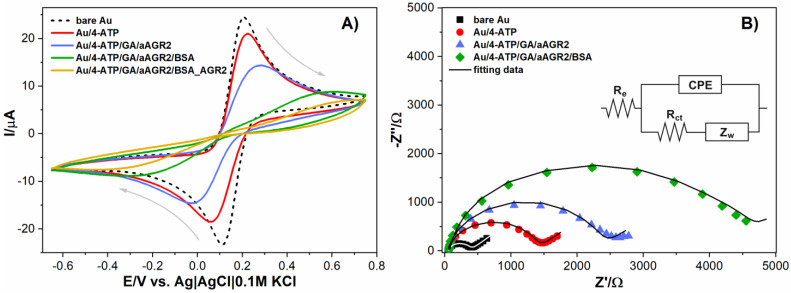
CV (**A**) and EIS (**B**) plots recorded for bare and modified Au electrode in 1 mM Fe(CN)_6_^3−/4−^/0.1 M PBS with scan rate 50 mV/s.

For the electrochemical measurements, the CV was recorded before and after the deposition of the 4-ATP monolayer. In Figure 2, the Fe(II)/Fe(III) redox peaks exhibit decreased signals after the functionalization of the 4-ATP monolayer. This can be attributed to a decrease in the electron transfer rate that was created by the compactness of the formulated SAMs. After antibody binding, the redox peaks decreased even more due to the decrease in the electron transfer rate. This was due to an increase in the biolayer thickness that was developed on gold surface. The cyclic voltammograms are strongly affected by the deposited layers, the difference between the anodic and cathodic peak potentials does not remain constant, whereas the peak current is modified significantly. The initial characteristic quasi-reversible redox cycle for a bare gold electrode can be seen. After its functionalization with BSA, the electron transfer between the redox probe and electrode surface was severely affected and an obvious decrease of the anodic and cathodic peaks was observed.

After anti-AGR2 IgG immobilization on the functionalized electrode surface, the peak currents of the redox couple of ferricyanide/ferrocyanide decrease. Immunochemical reaction of protein AGR2 molecules with the antibody film revealed a decrease in the Faradaic response. An increase was observed in the peak-to-peak separation between the cathodic and anodic waves of the redox probe, indicating that the electron-transfer kinetics of ferricyanide/ferrocyanide was obstructed.

**Figure 3:**
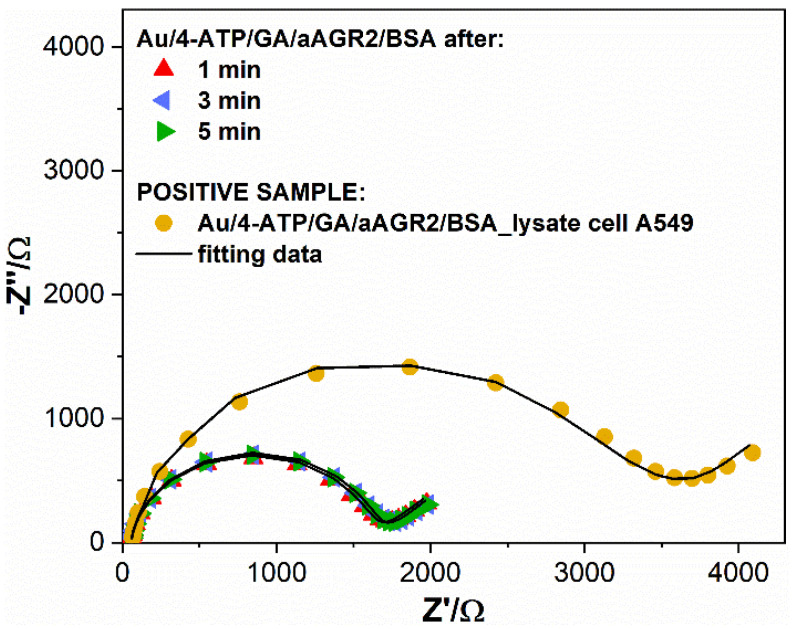
EIS measurement of biosensor response in time, after BSA modification. Registered in 1 mM K_3_[Fe(CN)_6_]/K_4_[Fe(CN)_6_]/0.01 M PBS.

Figure 2B and Figure 3A show the impedance spectra recorded with the EIS technique for a pure gold electrode and after each stage of the modification of its surface and during the detection of the AGR2 protein, respectively. The shape of the impedance spectra in the tested measuring frequency range from 100 kHz to 0.1 Hz is identical. The EIS spectra consists of one time constant. In the range of the highest and intermediate measurement frequencies, there is a capacitive loop, while at the lowest measurement frequencies, its presence is manifested by the Warburg impedance associated with diffusion control (straight line inclined at an angle of 45° to the *X*-axis).

For the analysis of the impedance spectra, the electrical equivalent circuit represented by the Randles circuit R_e_(CPE[R_ct_W]) [35] was used. R_e_ is the electrolyte resistance, CPE is the constant phase element (represented by Q and n), R_ct_ is the charge transfer resistance, and W is the Warburg impedance. The electrical equivalent circuit in the form of a Randles circuit is one of the basic models for describing the electrochemical processes occurring at the electrode/electrolyte interface. It is also widely used for impedance data analysis in electrochemical sensor/biosensor research [33,36,37,38].

Comparing the spectra recorded for bare screen-printed gold electrode, antibody-modified surface, and after saturation with a BSA solution, we observed a significant increase in resistance R_ct_ values. Such an increase in R_ct_ suggests successful binding between antigen and bare gold surface followed by unspecific binding of BSA to the modified gold substrate.

Stability analysis for the fully functionalized sensor can be seen in Figure 3. The BSA concentration was kept in the same order of magnitude to receive comparable results and impedance spectra were recorded in time. The sensor response was checked in time cycles of 1, 3, and 5 min.

### 3.2. Impedance Measurements for the Detection of the AGR2 Protein

**Figure 4:**
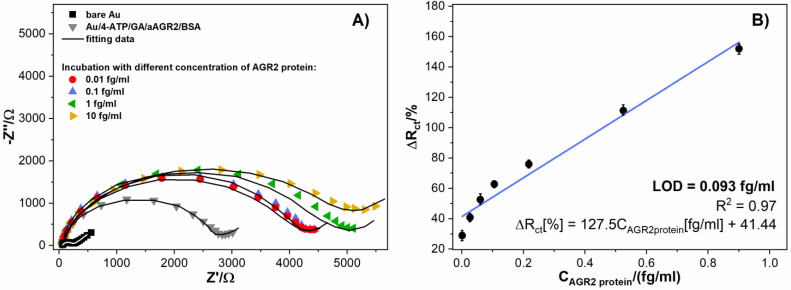
(**A**) Impedance spectra of Au/4-ATP/GA/aAGR2/BSA electrode after incubating in solutions with different protein concentrations recorded in 1 mM K_3_Fe(CN)_6_/K_4_[Fe(CN)_6_]/0.01 M PBS, (**B**) The relation between the sensor response expressed as R_ct_ change (ΔR_ct_) and the protein concentration. Registered in 1 mM K_3_[Fe(CN)_6_]/K_4_[Fe(CN)_6_]/0.01 M PBS. Error bars denote confidence interval (α = 0.05, n = 3).

The next stage of verification of the modified screen-printed gold electrode as a potential biosensor includes subsequent incubation in diluted solution of AGR2 protein and recording the electrode response with electrochemical impedance spectroscopy (Figure 4A). Here, the charge transfer resistance (R_ct_) increases gradually as the protein concentration increases after consecutive incubations. This was made from 0.01 fg/mL to 1 fg/mL. Table 1 presents the resistance values with incubation.

Figure 4B displays the impedance changes for the developed immunosensor incubated with different AGR2 protein concentrations. The tested gold impedance immunosensor was characterized by a linear response from 0.001 fg/mL to 0.900 fg/mL. The linear regression equation can be expressed as ΔR_ct_[%] = 127.5 C_AGR2protein_[fg/mL] + 41.44 with a correlation coefficient of R^2^ = 0.97. The values of relative standard deviation were calculated and ranged from 4.3% to 6.9%. The sensitivity is 127.5%(fg/mL)^−1^. The obtained limit of detection (LOD) for the presented immunosensor equals 0.093 fg/mL (S/N = 3). Linearity ranges and LODs of different methods for AGR2 protein detection are presented and compared in Table 2.

After the confirmation of the IgG anchor and AGR2 protein detection onto the gold surface, the transferability of the proposed substrate was examined on a biological sample where AGR2 is present at low levels in crude lysates. For this experimental step, lysate from A549 cells was chosen, which expresses AGR2 protein [35]. Negative controls of PBS and medium containing lysis buffer were also evaluated. The samples’ incubations on the Au/4-ATP/GA/aAGR2/BSA modified electrodes lasted 5 min and the unbound particles were flushed away with deionized water. Next, the EIS spectra were recorded. For each sample, a separate substrate was used. The complex detection with a mix of both positive and negative samples on the same electrode was also examined. 

**Figure 5:**
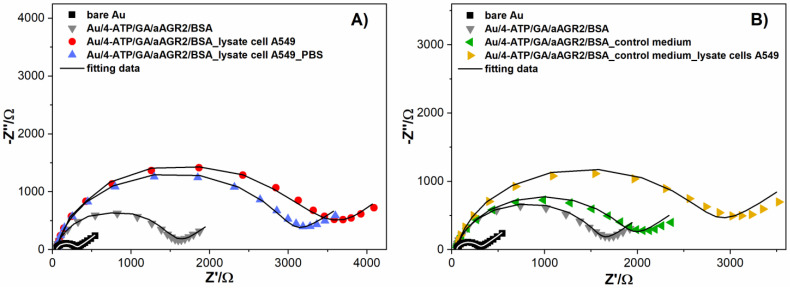
Nyquist plots of impedance spectra for bare and modified Au electrodes in 1 mM K_3_Fe(CN)_6_/0.1 M PBS at frequencies ranging between 100 Hz and 0.1 kHz. (**A**) electrode after incubating in solution with lysate cell A549 and PBS (**B**) electrode after incubating in solution with control medium and lysate cell A549.

No crucial changes were observed for separate measurements of PBS and control medium. The electrodes’ impedimetric characteristic stayed practically unchanged (Figure 5). For the measurements of the mix of negative samples and lysate, representative data are presented in Figure 5A, which shows impedance changes of the gold electrode during the modification steps of the sensor and antigen detection. The substantial impedance increase was observed after anti-AGR2 antibodies were linked and BSA free-sites were blocked, confirming the presence of a densely packed protein biolayer. The incubation of the Au/4-ATP/GA/aAGR2/BSA modified electrode with cell lysate A549 resulted in successive impedance increases compared to the ready biosensor (Figure 5B). After the AGR2 protein detection from the lysate, the same electrode was flushed with water and incubated with PBS buffer to exclude unspecific interactions. The impedance decreased slightly, which might be attributed to some disorder of the structure during electrode displacement and rinsing. Most importantly, the R_ct_ did not increase after incubation with PBS, which would indicate false positive antibodies–antigen interactions.

As further controls, Figure 5B highlights the impedance changes of the gold electrode during modification steps of the sensor and antigen detection. This time, after proper modification steps, the BSA saturated electrode was incubated with a negative control– medium. An increase in impedance was observed, however negligible, compared to the detection of the AGR2 protein in the lowest concentration of 0.01 fg/mL (Figure 5B). The R_ct_ value increased 57% (from 942.41 Ω to 1667.6 Ω). On the second step, the electrode was rinsed with water and incubated again, this time with A549 cell lysate. The above results confirmed the specificity of the developed sensor in the presence of negative buffers and positive cell lysate samples. The detailed parameters for both sensors (Figure 5A and Figure 4B) with the chosen equivalent circuit were summarized in Table 1.

### 3.3. Biosensor Selectivity, Repeatability and Stability Studies

**Figure 6:**
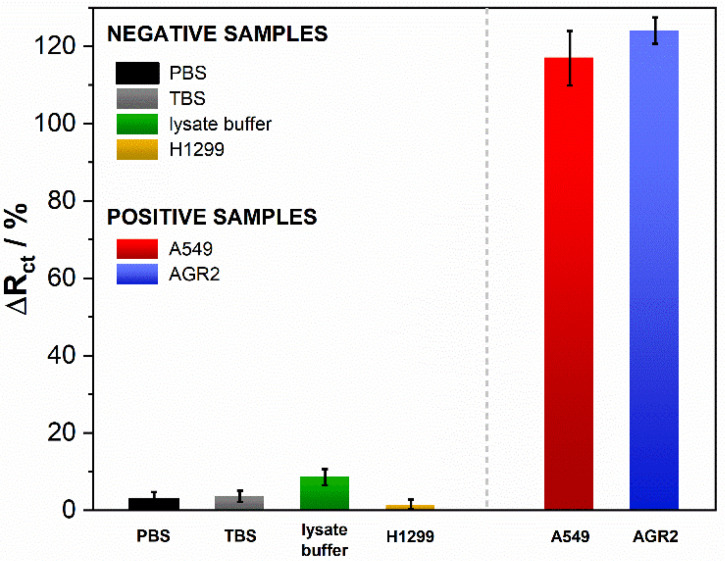
The plot of R_ct_ percentage changes as a response of the biosensor after incubation with negative and positive samples. The error bars show the standard deviations for three individual experiments.

PBS, TBS, lysate buffer, and H1299 cells were used as negative samples to investigate the selectivity of the immunosensor. As positive samples we used AGR2 protein and A549 cell lysates containing the AGR2 protein (Supplementary Figure S1). The negative samples’ concentration was kept in the same order of magnitude to receive comparable results. Next, they were applied to the modified electrode separately. After 5 min incubation, the EIS spectra were recorded. According to the assumptions, there was no significant increase in the value of R_ct_ parameter for the negative samples: PBS, TBS, lysate buffer, and H1299 cells. Additionally, these changes did not exceed 10% (Figure 6). Compared to the %R_ct_ change in the presence of positive AGR2 protein and A549 cells, the negative samples were proved to give no cross-reactivity, indicating that the proposed method has high selectivity for the detection of protein AGR2. The percentage of changes in the charge transfer resistance parameter for positive samples was noticeably larger and ranged from 117.11% to 124%. It can be concluded from the measurements that the antibodies used in these studies properly bind to each species of pathogen for which the sensor was designed. All measurements were repeated on a series of three electrodes to confirm the lack of influence of negative samples on further measurements. Furthermore, the relative standard deviations (RSD) took values from 2 to 10%, which indicates the high stability of the proposed system.

## 4. Conclusions

In summary, we have reported the extended investigation of an impedimetric immunosensor for AGR2 protein detection. The mechanism is based on the EIS spectra recorded at antibody-modified gold electrodes. These data build on our prior research to develop a gold electrode platform [33] but in this report we apply this to a monoclonal antibody that targets a cancer antigen, AGR2. The obtained limit of detection (LOD) for the presented immunosensor equals 0.093 fg/mL (S/N = 3). The advantage of this ‘one-step’ diagnostic assay relative to an ELISA or mass spectrometry is rapid and sensitive measurement of antigen binding. This provides a proof of concept that we intend to use to develop clinical trials with plasma in order to determine whether AGR2 antigen detection in patient fluid provides any prognostic indicator.

## Figures and Tables

**Table 1:** The values of the elements obtained using EEQC—unmodified screen-printed gold electrode and screen-printed gold electrode modified with various concentration of protein.

Sample	R_e_ [Ω]	CPE [μΩ^−1^s^n^]	n	R_ct_ [Ω]	AW [Ωs^−0.5^]
bare Au	50.058	2.62	0.915	315.57	3.893
Au/4-ATP/GA/aAGR2	49.896	0.90	0.951	2223.9	701.63
Au/4-ATP/GA/aAGR2/BSA	53.244	0.98	0.939	2259.2	824.3
Au/4-ATP/GA/aAGR2/BSA_protein AGR2 0.01 fg/mL	48.971	1.06	0.920	3449.3	1266.8
Au/4-ATP/GA/aAGR2/BSA_protein AGR2 0.1 fg/mL	50.617	1.09	0.920	3704.4	1308.4
Au/4-ATP/GA/aAGR2/BSA_protein AGR2 1 fg/mL	67.882	1.18	0.920	4887.6	1658.3
Au/4-ATP/GA/aAGR2/BSA_protein AGR2 10 fg/mL	68.882	1.20	0.920	5790,9	1667.6

**Table 2:** The comparison of AGR2 proteindetection methods using electrochemical techniques on the various substrate.

Detection Substrate	Target Molecules	Turnaround Time	Sensitivity	Limit of Detection	Reference
ITO (CV, DPV, EIS)	CA15-3	15 min	13 µL^−1^ng/cm^−2^	0.001 ng/mL	[39,40]
FTO (CV)	plasminogen activator receptor	35 s	-	4.8 fM	[40,41]
Polypyrrole-gold nanocomposite (DPV)	CA125	80 min	-	30.9 ng/mL	[42]
AuSPE (EIS, DPV)	MCF-7	91 s	77 EVs/mL	77 particles/mL	[40,43]
SPCE (CV, EIS)	MUC1	45 min	-	0.02 U/mL	[40,44]
GCE (DPV, Polylysine modification)	AGR2	90 min	-	2.3 fM	[40,45]
GE (DPV, AgNPs modification)	AR-42	90 min	-	6 cells/mL	[40,46]
Au (EIS)	AGR2	3 min	127.5%(fg/mL)^−1^	0.093 fg/mL	this work

## Supplementary Materials

The following are available online at https://www.mdpi.com/article/10.3390/bios11060184/s1, Figure S1: Immunohistochemical expression of AGR2 protein in A549 cells. The A549 cells were fixed with 4% (*v*/*v*) paraformaldehyde in PBS at RT for 15 min, washed with PBS three times, and permeabilized using 0.25% triton X-100 in PBS at RT for 10 min. Then, the cells were again washed with PBS three times and blocked with 3% BSA in PBS for 1 h. The primary antibody was incubated at appropriate dilution (typically 1:1000) overnight at 4 °C. Alexa Fluor 488 goat anti-mouse (Invitrogen, USA) secondary antibody was incubated at RT for 1 h. Coverslips were washed three times with PBS in between each step. Cells were incubated in DAPI (image B and C) (Invitrogen, USA) diluted at 1:10,000 with dH2O for 5 min to stain the nucleus. An additional 3 washes with dH2O for 5 min were performed. A single drop of Fluorescence Mounting Medium (S3023, Dako, Denmark) was used to mount the cells on the slide. The fluorescent signal (image A and C) was detected using a Zeiss Axioplan 2 microscope (63× or 100× oil immersion objective). Images were acquired by Micro-Manager 1.4 software. Images were processed in ImageJ 2.0 software.
