# Supplementary material for: An Ultrasensitive Biosensor for Detection of Femtogram Levels of the Cancer Antigen AGR2 Using Monoclonal Antibody Modified Screen-Printed Gold Electrodes
Source: Biosensors (Basel). 2021 Jun 7;11(6):184. doi: 10.3390/bios11060184 (PMC8230265; doi:10.3390/bios11060184)
Supplement: Supplementary file 1 [file biosensors-11-00184-s001.zip › biosensors-1194575-supplementary.pdf]

An Ultrasensitive Biosensor for Detection of Femtogram Levels of the Cancer Antigen AGR2 Using Monoclonal Antibody Modified Screen-Printed Gold Electrodes

Wioleta Białobrzeska [1,*], Karolina Dziąbowska [2], Małgorzata Lisowska [3], M. Aiman Mohtar [4], Petr Muller [5], Borivoj Vojtesek [5], Radovan Krejcir [5], Robert O' Neill [6], Ted R. Hupp [3,7], Natalia Malinowska [1], Ewelina Bięga [1], Daniel Bigus [1], Zofia Cebula [1], Katarzyna Pala [2], Elżbieta Czaczyk [2], Sabina Żołędowska [1] and Dawid Nidzworski [1]

[1] Institute of Biotechnology and Molecular Medicine, 3 Trzy Lipy St., 80-172 Gdansk, Poland; n.malinowska@ibmm.pl (N.M.); e.biega@ibmm.pl (E.B.); d.bigus@ibmm.pl (D.B.); z.cebula@ibmm.pl (Z.C.); sabina.zoledowska@etongroup.eu (S.Ż.); dawid@etongroup.eu (D.N.)

[2] SensDx, 14b Postępu St., 02-676 Warszawa, Poland; karolina.dziabowska@gmail.com (K.D.); katarzyna.pala@etongroup.eu (K.P.); ela@etongroup.eu (E.C.)

[3] International Centre for Cancer Vaccine Science, University of Gdansk, Kładki 24 St., 80-822 Gdańsk, Poland; malgorzata.lisowska@ug.edu.pl (M.L.); ted.hupp@ed.ac.uk (T.R.H.)

[4] UKM Medical Centre, UKM Medical Molecular Biology Institute (UMBI), Universiti Kebangsaan Malaysia, 56000 Cheras, Kuala Lumpur, Malaysia; m.aimanmohtar@ppukm.ukm.edu.my

[5] Research Centre for Applied Molecular Oncology, Masaryk Memorial Cancer Institute, 65653 Brno, Czech Republic; pmuller@post.cz (P.M.); vojtesek@mou.cz (B.V.); radovan.krejcir@mou.cz (R.K.)

[6] Cambridge Oesophagogastric Centre, Cambridge University Hospitals NHS Foundation Trust, Cambridge CB2 0QQ, UK; robertoneill@nhs.net

[7] Institute of Genetics and Molecular Medicine, University of Edinburgh, Edinburgh, EH4 2XR, UK

* Correspondence: w.bialobrzeska@ibmm.pl

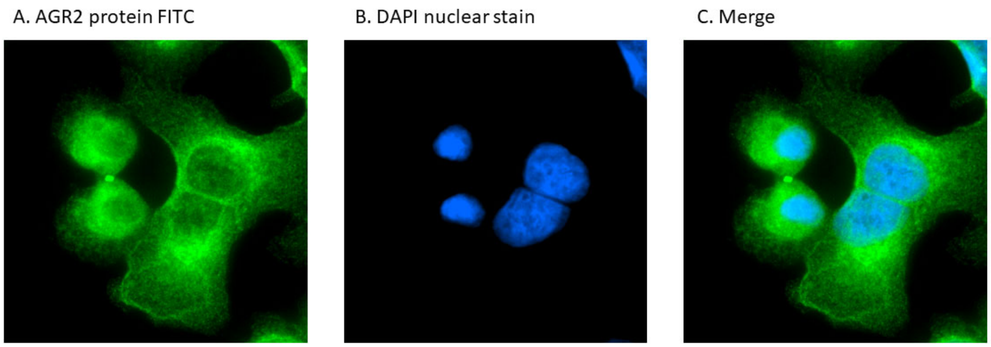

Figure S1. Immunohistochemical expression of AGR2 protein in A549 cells. The A549 cells were fixed with 4% (v/v) paraformaldehyde in PBS at RT for 15 min, washed with PBS three times, and permeabilized using 0.25% triton X-100 in PBS at RT for 10 min. Then, the cells were again washed with PBS three times and blocked with 3% BSA in PBS for 1 h. The primary antibody was incubated at appropriate dilution (typically 1:1000) overnight at 4°C. Alexa Fluor 488 goat anti-mouse (Invitrogen, USA) secondary antibody was incubated at RT for 1 h. Coverslips were washed three times with PBS in between each step. Cells were incubated in DAPI (image B and C) (Invitrogen, USA) diluted at 1:10,000 with dH_2O for 5 min to stain the nucleus. An additional 3 washes with dH_2O for 5 min were performed. A single drop of Fluorescence Mounting Medium (S3023, Dako, Denmark) was used to mount the cells on the slide. The fluorescent signal (image A and C) was detected using a Zeiss Axioplan 2 microscope (63x or 100x oil immersion objective). Images were acquired by Micro-Manager 1.4 software. Images were processed in ImageJ 2.0 software.
